# Synergy between “Probiotic” Carbon Quantum Dots and Ciprofloxacin in Eradicating Infectious Biofilms and Their Biosafety in Mice

**DOI:** 10.3390/pharmaceutics13111809

**Published:** 2021-10-29

**Authors:** Yanyan Wu, Guang Yang, Henny C. van der Mei, Linqi Shi, Henk J. Busscher, Yijin Ren

**Affiliations:** 1 University of Groningen and University Medical Center of Groningen, Department of Orthodontics, Hanzeplein 1, 9700 RB Groningen, The Netherlands; y.wu@umcg.nl (Y.W.); y.ren@umcg.nl (Y.R.); 2 State Key Laboratory of Medicinal Chemical Biology, Key Laboratory of Functional Polymer Materials, Ministry of Education, Institute of Polymer Chemistry, College of Chemistry, Nankai University, Tianjin 300071, China; 18202265300@163.com (G.Y.); lqshi@nankai.edu.cn (L.S.); 3 University of Groningen and University Medical Center Groningen, Department of Biomedical Engineering, Antonius Deusinglaan 1, 9713 AV Groningen, The Netherlands; h.j.busscher@umcg.nl

**Keywords:** matrix disruption, antibiotics, *Bifidobacterium breve*, *Escherichia coli*, reactive oxygen species, intestinal infections, biosafety

## Abstract

Orally administrated probiotic bacteria can aid antibiotic treatment of intestinal infections, but their arrival at their intestinal target site is hampered by killing in the gastrointestinal tract and by antibiotics solely intended for pathogen killing. Carbon-quantum-dots are extremely small nanoparticles and can be derived from different sources, including bacteria. Here, we hypothesize that carbon-quantum-dots inherit antibacterial activity from probiotic source bacteria to fulfill a similar role as live probiotics in intestinal infection therapy. Physico-chemical analyses indicated that carbon-quantum-dots, hydrothermally derived from *Bifidobacterium breve* (B-C-dots), inherited proteins and polysaccharides from their source-bacteria. B-C-dots disrupted biofilm matrices of *Escherichia coli* and *Salmonella typhimurium* biofilms through extensive reactive-oxygen-species (ROS)-generation, causing a decrease in volumetric bacterial-density in biofilms. Decreased bacterial densities leave more open space in biofilms and have enhanced ciprofloxacin penetration and killing potential in an *E. coli* biofilm pre-exposed to probiotic B-C-dots. Pathogenic carbon-quantum-dots hydrothermally derived from *E. coli* (E-C-dots) did not disrupt pathogenic biofilms nor enhance *E. coli* killing potential by ciprofloxacin. B-C-dots were biosafe in mice upon daily administration, while E-C-dots demonstrated a decrease in white blood cell and platelet counts and an increase in C-reactive protein levels. Therefore, the way is paved for employing probiotic carbon-quantum-dots instead of viable, probiotic bacteria for synergistic use with existing antibiotics in treating intestinal infections.

## 1. Introduction

The ongoing increase in the number of antimicrobial-resistant bacterial strains has made the treatment of infectious biofilms across the human body extremely difficult, and in many cases, antibiotics have lost a considerable part of their usefulness [1,2,3]. At the same time, the development of new antibiotics is stalling due to high costs and poor pros-pects of an industrial return of investment, given the short time between market introduction and the occurrence of resistance against a new antibiotic. Probiotic bacteria promote health and can aid in controlling infectious bacteria through a variety of mechanisms, such as prevention of pathogen adhesion, production of bacteriocins, generation of reactive oxygen species (ROS), downregulation of toxin production in pathogens, nutrient competition, competitive adhesion and pathogen exclusion from surfaces and immune modulation [4,5,6,7,8]. Probiotic bacteria are predominantly administered orally to assist the development of a healthy intestinal microflora, but their arrival at their intestinal target site is greatly hampered by killing in the low pH environment of the gastrointestinal tract [9,10]. Recently, a combination of probiotic bacteria with existing antibiotics has been demonstrated to synergistically increase antibiotic efficacy against infectious biofilms [11]. Unfortunately, in the clinical scenario, this synergy is difficult to exploit because probiotic bacteria may not only be killed by acidic gastric fluid but also by the antibiotics with which they are co-administered.

Nanotechnology has offered a number of new nano-antimicrobials, including carbon quantum dots (C-dots). C-dots possess diameters less than 10 nm and have good photostability combined with low toxicity [12,13]. C-dots are easily synthesized from a wide variety of carbon sources, which include spermidine [14], gentamicin sulfate [15], vitamin C [16], carbon nanopowders [17], cigarette smoke [18], polyethyleneimine and citric acid [19] and bacteria [20,21,22,23]. Interestingly, C-dots synthesized from antibiotics, and antimicrobial ammonium salts can inherit chemical functionalities from their carbon sources, yielding C-dots with antibacterial activity against a broad spectrum of Gram-positive and Gram-negative pathogens [15,24,25]. C-dots derived from *Lactobacillus plantarum* were found to reduce adhesion and subsequent growth of an *Escherichia coli* biofilm in a prophylactic mode of use [20].

Here we hypothesize, that C-dots synthesized from probiotic bacteria as a carbon source may inherit antibacterial activity from probiotic source strains that make them suitable as a non-viable alternative for synergistic use with antibiotics for the treatment of infectious intestinal biofilms (therapeutic mode of use). When proven that “probiotic” C-dots indeed possess probiotic properties with synergistic antibacterial activity with antibiotics, the oral administration of probiotic C-dots constitutes a simple solution for the problems associated with the oral administration of probiotic bacteria.

This paper aims to prove the above hypothesis. To this end, “probiotic” C-dots hydrothermally derived from *Bifidobacterium breve* will first be evaluated for their ability to disrupt existing infectious biofilms of three pathogenic *E. coli* strains and a *Salmonella* *typhimurium* strain (all common intestinal pathogens). Next, an existing *E. coli* biofilm will be pre-exposed to probiotic C-dots, after which the disrupted biofilm will be exposed to ciprofloxacin, a commonly used antibiotic. Biosafety of probiotic C-dots will be established in an in vivo murine model. “Pathogenic” C-dots derived from a pathogenic *E. coli* strain will be included for comparison in order to establish whether antibacterial activity can be uniquely inherited by C-dots hydrothermally carbonized from probiotic bacteria or also when carbonized from a pathogen.

## 2. Materials and Methods

### 2.1. Bacteria Growth Conditions and Harvesting

All strains except *E. coli* Hu 734 were purchased from the American Type Culture collection (ATCC, Manassas, VA, USA). *E. coli* Hu 734 was a human clinical isolate. *B. breve* ATCC 15700 was grown anaerobically on a Reinforced Clostridial Medium (RCM, Becton & Dickinson, Le Pont-de-Claix, France) agar plate at 37 °C. Different *E. coli* strains and *S. typhimurium* were grown aerobically on blood agar plates at 37 °C. One colony of *E. coli*, *B. breve* or *S. typhimurium* was inoculated into Brain Heart Infusion (10 mL, BHI, OXOID, Basingstoke, UK), RCM (10 mL) or Nutrient Broth (NB, OXOID), respectively, and incubated for 24 h under the appropriate conditions. This preculture was transferred to 200 mL liquid medium and grown for 18 h. Bacterial cultures were harvested by centrifugation for 5 min at 5000× *g*, washed twice with demineralized water and resuspended in water. Washed bacterial pellets were collected in 15 mL centrifuge tubes, frozen in liquid nitrogen and subsequently freeze-dried (Edwards freeze dryer, Richmond Scientific, Chorley, UK) for FTIR and XPS. For other experiments, the resulting bacterial suspensions were sonicated for 30 s, while cooling in an ice/water bath to remove bacterial aggregates. The bacteria were enumerated using a Bürker-Türk counting chamber and resuspended in 10 mM potassium phosphate buffer (pH 7.4) to a different concentration for the different experiments.

### 2.2. Preparation and Characterization of Carbon Quantum Dots

Bacterial-derived C-dots were prepared according to a previously reported hydrothermal method [20]. Briefly, *B. breve* ATCC 15700 was harvested from a 200 mL culture and *E. coli* ATCC 25922 was harvested from a 400 mL culture and they were washed as described above and resuspended in 40 mL of demineralized water. The bacterial suspensions were put in a 100 mL Teflon-lined stainless-steel autoclave and heated for 24 h at 200 °C. In order to remove large C-dots, the brown solution was centrifuged at 15,800× *g* for 15 min. For further removal of large C-dots, the supernatant was filtered through a 0.22 µm filter membrane and dialyzed against demineralized water (cut-off 1 kDa) for 2 days, while stirring at room temperature and refreshing the water 3 times a day. The bacterial-derived C-dots were freeze-dried and stored at −20 °C.

C-dots were imaged using Transmission Electron Microscopy (TEM). The C-dots in demineralized water were put onto glow discharge-treated carbon-coated copper grids. The excess suspension was removed by touching the grid edge to filter paper. The C-dots on the carbon-coated grid were stained with 2% uranyl acetate solution, and the excess stain was removed [26]. TEM images were taken using an electron microscope operating at 120 kV (Philips CM 120, Eindhoven, The Netherlands). In addition, zeta potentials of the C-dots (500 µg/mL) were measured in 10 mM potassium phosphate buffer at pH 7.4 using dynamic light scattering at 25 °C (ZetaSizer, Malvern Instruments, Malvern, UK).

FTIR spectra (Cary 600 series, Agilent Technologies, Santa Clara, CA, USA) were taken from freeze-dried bacteria and C-dots by mixing the bacteria and C-dots with KBr and pressing into a KBr-tablet [27]. FTIR spectra were recorded over the wavenumber range of 4000 to 400 cm^−1^ with a resolution of 4 cm^−1^. A total of 32 scans were taken and averaged. The areas of the most important absorption bands were determined by integration after linear background subtraction and normalized with respect to the area of the CH stretch absorption region around 2930 cm^−1^ [27].

The elemental surface composition of freeze-dried bacteria and C-dots were determined using X-ray Photoelectron Spectroscopy (XPS, S-probe, Surface Science Instruments, Mountain View, CA, USA), operated at a vacuum of 10^−9^ Pa. The X-ray (10 kV, 22 mA) beam was produced using an aluminum anode and had a spot size of 250 × 1000 μm. The freeze-dried bacteria and C-dots were pressed in small stainless steel cups and put in the XPS chamber [28]. For overall spectra, scans were made in the binding energy range of 1–1200 eV at low resolution. For C_1s_ narrow scans, high-resolution scans were recorded over a 20 eV binding energy range. The C_1s_ binding energy was set at 284.8 eV. The area under each peak, after background subtraction, was used for the calculation of peak intensities, yielding elemental surface concentrations.

UV-vis absorption spectra were obtained in a quartz cuvette using a UV-vis spectrophotometer (5082 PerkinElmer Lambda 2, Waltham, MA, USA). Fluorescence emission spectra from the C-dots were measured using a spectrofluorometer (Jasco FP-8300, Tokyo, Japan). C-dots were suspended in 10 mM potassium phosphate buffer (pH 7.4) to an absorbance intensity below 0.1. The same suspensions were used to measure the quantum yield using quinine sulfate (0.1 M H_2_SO_4_; quantum yield 54%) as a standard [29].

Subsequently, the quantum yield of the C-dots was calculated as

(1) $$\Phi_{x}=\Phi_{st}\left(\frac{K_{x}}{K_{st}}\right){\left(\frac{\eta_{x}}{\eta_{st}}\right)}^{2}$$

in which Φ*_x_* and Φ*_st_* are the quantum yields of the nanoparticles to be determined and the standard applied, respectively, *K_x_* and *K_st_* are the slopes of a linear regression on the absorbance as a function of the integrated photoluminescence and *η_x_* and *η_st_* are the refractive indices of the nanoparticle suspension and the standard solution, respectively.

### 2.3. Minimum Inhibitory Concentration (MIC) and Minimal Bactericidal Concentration (MBC) of Carbon Quantum Dots

To determine the MIC of bacterially-derived B-C- and E-C-dots (initial concentration 2000 µg/mL), 100 µL was serially diluted into BHI for *E. coli* strains or NB for *S. typhimurium* in a 96-wells plate, after which *E. coli* ATCC 25922, *E. coli* ATCC 8739, *E. coli* Hu 734 or *S. typhimurium* ATCC 14028 (100 μL of 2 × 10^5^ bacteria/mL), suspended in a growth medium, were added. After incubation for 24 h at 37 °C, the MIC values were taken as the lowest C-dot concentration at which bacterial growth was not visible. Subsequently, the MBC values were determined by plating aliquots of the bacterial suspensions yielding no visible growth after 24 h on BHI or NB agar plates. After 24 h incubation at 37 °C, the lowest concentration at which colony formation remained absent was taken as the MBC. The MIC and MBC of the different *E. coli* strains were also determined against ciprofloxacin as described above.

### 2.4. Biofilm Exposure to B-C- and E-C-Dots

Suspensions of 1 mL of the different *E. coli* strains or the *S. typhimurium* strain (1 × 10^9^ bacteria/mL) in 10 mM potassium phosphate buffer at pH 7.4 were added to a 12-wells plate and left for 2 h at 37 °C to allow bacteria to adhere. After 2 h, the bacterial suspension was removed, and the well was washed once with 10 mM phosphate buffer. A total of 2 mL growth medium was added to each well, and bacteria were incubated at 37 °C. After 24 h, the growth medium was removed, and the biofilm was washed once with 10 mM potassium phosphate buffer pH 7.4. The biofilms were exposed to different concentrations of B-C- and E-C-dots (125, 250, 500 µg/mL) in 10 mM potassium phosphate buffer pH 7.4 for 4 h, after which the C-dot suspensions were carefully removed, and the biofilms were washed twice with phosphate buffer.

For confocal laser scanning microscopy (CLSM) (Leica TCS SP2 Leica, Germany), the biofilms were stained with LIVE/DEAD stain (*Bac*Light^TM^, Molecular probes, The Netherlands) containing SYTO9 and propidium iodide for 20 min in the dark. After 20 min, the stain was removed, and the biofilms were immersed in 10 mM phosphate buffer (pH 7.4) and imaged using CLSM. An argon ion laser at 488 nm was used to excite SYTO9 (green fluorescent), and a HeNe laser at 543 nm was used to excite propidium iodide (red fluorescent), collecting fluorescence at 500−535 nm (SYTO9) and 583−688 nm (propidium iodide). The biofilm images were further analyzed using ImageJ and COMSTAT to determine biofilm thickness and the percentage of live/dead bacteria [30]. After taking confocal images, the biofilms were sonicated for 30 s, and the total number of bacteria (live and dead) per biofilm was derived using a Bürker-Türk counting chamber, from which the bacterial density in the biofilm was determined.

### 2.5. Combined Effects of Pre-Exposure of Biofilms to Carbon Quantum Dots Followed by Antibiotics

A total of 100 µL of *E. coli* suspension (1 × 10^9^ bacteria/mL) in 10 mM potassium phosphate buffer at pH 7.4 was added to a 96-wells plate and left for 2 h to allow bacterial adhesion at 37 °C. After 2 h, the bacterial suspension was removed, and the well was washed once with phosphate buffer. A total of 200 μL BHI was added to each well, and the bacteria were incubated at 37 °C. After 24 h, the medium was removed, and the biofilms were pre-exposed to B-C- or E-C-dots (200 μL of 500 µg/mL) in phosphate buffer for 4 h. After 4 h, the C-dot suspensions were removed, biofilms were washed twice with phosphate buffer and ciprofloxacin in BHI medium was added for subsequent growth in the presence of different concentrations (0, 2, 10, 20 times MBC, see Table S1) of antibiotics for 24 or 72 h. The growth medium with ciprofloxacin was refreshed every 24 h by removal with a pipet and slowly refilled by pipetting fresh medium with ciprofloxacin against the wall of the well. After ciprofloxacin exposure, biofilm bacteria were removed and suspended in 10 mM phosphate buffer (pH 7.4). The number of CFUs per biofilm was determined by serial dilutions and plated on the BHI agar plates. The CFUs were counted after a 24 h incubation at 37 °C.

### 2.6. Biosafety Assay in Mice

Seven–eight-week-old BALB/c mice were obtained from Vital River Laboratory Animal Technology Co. (Beijing, China). All animal experiments were approved by the Institutional Animal Care and Use Committee of Nankai University, Tianjin, China. The mice were divided into four groups of three mice each: (1) mice gavaged daily with 100 µL phosphate buffered saline (control), (2) mice gavaged with 100 µL of a suspension of commercially available activated charcoal (Aladdin Bio-Chem Technology) in phosphate buffered saline (positive control), (3) mice gavaged with 100 µL E-C-dots in phosphate buffered saline and (4) mice gavaged with 100 µL B-C-dots in phosphate buffered saline. The dosing was based on the recommendation for the commercial activated charcoal product after weight correction for the average body weight of mice and humans, yielding a dose of 0.3 mg/mouse/day. The administration was performed with a gavage needle over a 14-day period during which body weight and temperature were measured daily. On day 14, blood samples were collected from each mouse before sacrifice for routine analysis, while after sacrifice by cervical dislocation, the heart, liver, spleen, lungs and kidneys of mice were surgically excised, fixed in 10% formaldehyde, embedded into paraffin, cut in sections and stained with hematoxylin and eosin (H&E) for histological analyses.

### 2.7. Statistical Analyses

Differences were compared for statistical significance using one-way ANOVA. Differences were considered significant if *p* < 0.05. Statistical analysis was performed using GraphPad version 7.00 (GraphPad Software, La Jolla, CA, USA).

## 3. Results and Discussion

### 3.1. Physico-Chemical Characterization of Hydrothermally Derived C-Dots and Their Source Bacteria

**Figure 1:**
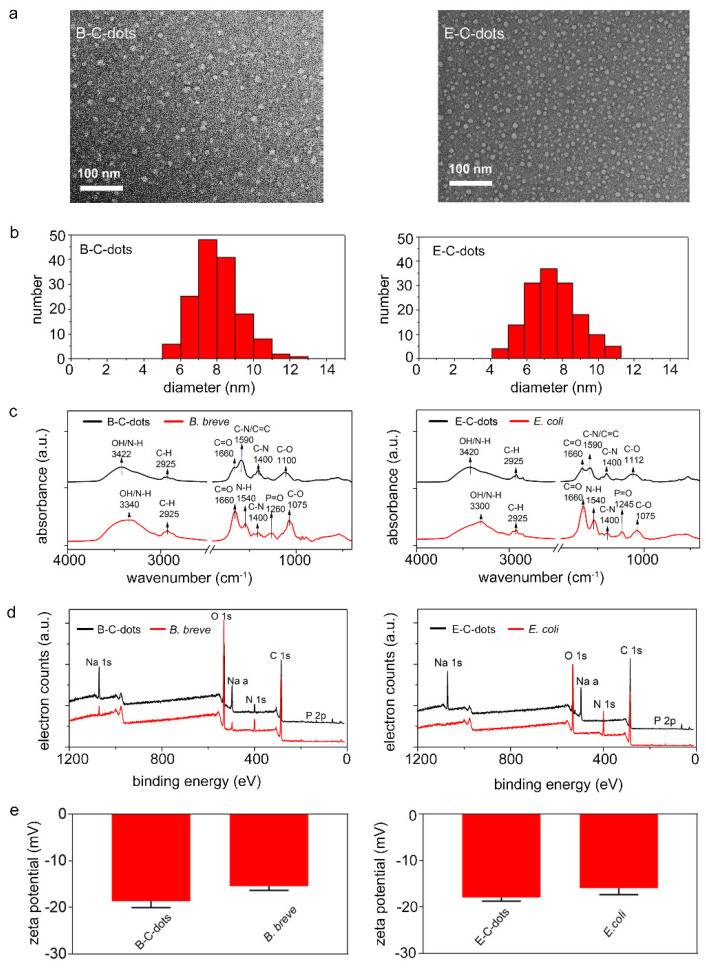
Physico-chemical characterization of C-dots and bacteria used for their hydrothermal carbonization. (**a**) TEM micrographs of C-dots derived from *B. breve* ATCC 15700 (B-C-dots) and *E. coli* ATCC 25922 (E-C-dots). (**b**) Diameter distributions of B-C- and E-C-dots as derived from TEM micrographs. Each distribution includes 150 C-dots. (**c**) FTIR spectra of *B. breve* and B-C-dots, *E. coli* and E-C-dots. Spectra have been baseline adjusted using Spectragryph software. (**d**) XPS spectra of B-C-dots and *B. breve*, E-C-dots and *E. coli*. (**e**) Zeta potentials of B-C-dots and *B. breve*, E-C-dots and *E. coli* were measured in 10 mM potassium phosphate (pH 7.4). Data are averages ± standard deviations over triplicate experiments with separately prepared nanoparticles and different bacterial cultures.

C-dots derived from probiotic *B. breve* (B-C-dots) and pathogenic *E. coli* (E-C-dots) sources both possessed spherical shapes (Figure 1a) with average diameters of 8.0 ± 1.3 and 7.5 ± 1.4 nm, respectively (Figure 1b). The composition of B-C- and E-C-dots were compared using Fourier transform infrared (FTIR) and X-ray photoelectron (XPS) spectroscopy. Comparisons also included spectroscopies on the freeze-dried bacteria employed for carbonization.

All FTIR spectra (Figure 1c), both of C-dots and their source bacteria, demonstrated a broad absorption band from 3600 to 2700 cm^−1^ originating from O–H and N–H stretching vibrations and a C–H stretching vibration band between 2950 and 2850 cm^−1^ [20,21,27,31]. Amide I (1660 cm^−1^, due to C=O stretching vibrations) [27,31] and Amide II (1540 cm^−1^ due to N-H bending vibrations) [27,31] absorption bands attested to the presence of proteins, as did the absorption bands at 1590 (due to C–N/C=C vibration) [32,33] and 1400 cm^−1^ (C–N stretching vibrations) [27,31]. The absorption band around 1260 cm^−1^ (PI) was assigned to stretching vibrations in P=O as occurring in phosphodiester of nucleic acids, while the absorption bands between 1200 and 950 cm^−1^ (PII) were mainly due to the stretching vibrations of C–O–C in polysaccharides [34,35]. The above absorption bands have been demonstrated to be common in highly different bacterial strains and species, and differences amongst strains could be revealed by normalizing absorption band areas with respect to the absorption band area of the C–H stretching band (2950 to 2850 cm^−1^) [34,35]. A similar normalization has been applied to the FTIR spectra measured here for bacteria and bacterially-derived C-dots (Table 1). Carbonization of bacteria into C-dots led to a significant decrease in the AmI/C–H absorption band (1660 cm^−1^) ratio. The reduced AmII/C–H absorption band (1540 cm^−1^) was difficult to discern due to overlapping with a new band arising after carbonization at 1590 cm^−1^. For both bacterial strains, a new IR absorption band at 1590 cm^−1^ appeared upon carbonization, indicating the formation of new C–N/C=C bonds due to the heterocyclic aromatic amines formed during hydrothermal carbonization [36]. The phosphate absorption band (1260 to 1245 cm^−1^) ratios decreased for both strains upon carbonization, while polysaccharide absorption band (1200 to 950 cm^−1^) ratios remained similar. This implies that upon hydrothermal carbonization of bacteria, polysaccharides may have been retained on bacterially-derived C-dots.

**Figure 2:**
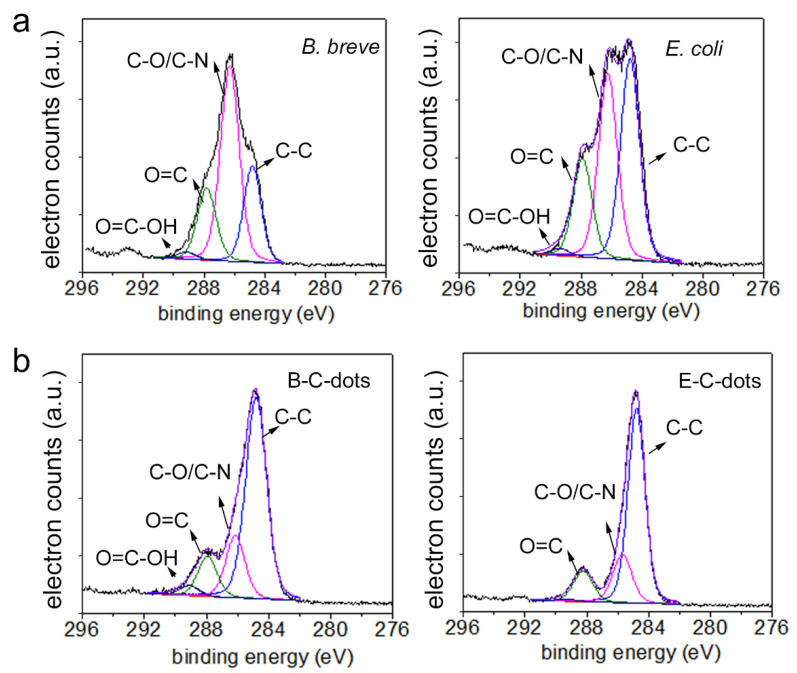
Narrow-scan C_1s_ binding energy spectra. (**a**) Spectra of *B. breve* ATCC 15700 and *E. coli* ATCC 25922 as carbon sources. (**b**) Spectra of B-C- and E-C-dots, as derived from the carbonization of *B. breve* and *E. coli*, respectively.

Moreover, XPS binding energy spectra were comparable to the XPS spectra found common for a wide variety of bacterial strains and species [37,38]. Qualitatively, the XPS spectra were similar for all C-dots and their source bacteria (Figure 1d). Oxygen in XPS spectra can be due to various components (proteins, phosphate and polysaccharides), while phosphorus is exclusively due to phosphate groups. Nitrogen in bacterial XPS spectra can be uniquely attributed to proteins, and quantitative analysis of the XPS spectra (see also Table 1) demonstrated a loss of proteins upon bacterial carbonization, concurrent with a decrease in the AmI/C–H absorption band area. This conclusion is also supported by a decrease in the at% of carbon involved in chemical functionalities representative of proteins (C_1s_ components at 286.1 and 288.0 eV) (Figure 2).

Zeta potentials of B-C- and E-C-dots were around −18 mV, which is slightly more negative than of their source bacteria possessing zeta potentials of around −15 to −16 mV (Figure 1e). Demonstration of a loss of proteins by bacteria upon protease treatment or spontaneous mutation has been demonstrated previously to be accompanied by less negative zeta potentials, in line with the current results [27,39]. Summarizing, C-dots hydrothermally derived from *B. breve* and *E. coli* inherit both proteins and polysaccharides from their source bacteria with accompanying zeta potentials. This is a different inheritance than obtained after pyrolytic carbonization of source bacteria in air through which polysaccharides were not retained in bacterially-derived C-dots [23]. Presumably, water bound to hydrophilic polysaccharides is preserved up to higher temperatures during hydrothermal carbonization than by carbonization in air, delaying the scission of characteristic polysaccharides to higher temperatures. This makes hydrothermal carbonization preferable above pyrolytic carbonization of bacteria for retaining chemical functionalities.

**Figure 3:**
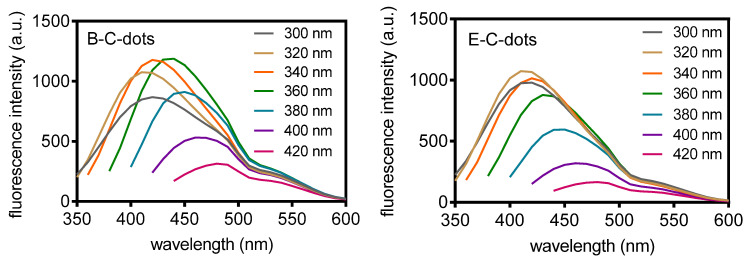
Fluorescence emission spectra of hydrothermally derived C-dots from *B. breve* ATCC 15700 (B-C dots) and *E. coli* ATCC 25922 (E-C-dots) at different excitation wavelengths (indicated in the panels) in 10 mM phosphate buffer (pH 7.4).

UV-vis absorption spectra of B-C- and E-C-dots both showed an absorption band at 270 nm, attributed to the π-π* transitions of conjugated C=C bonds (Figure S1) [14]. The absorption spectrum of B-C-dots showed a small shoulder band around 320 nm that was absent in E-C-dots, that can be ascribed to n-π* transitions of C–N and C=N bonds [40] and confirm a higher protein content of B-C-dots relative to E-C-dots (see also Table 1). Fluorescence emission spectra (Figure 3) were combined with absorption spectra to calculate the quantum yield of B-C-dots and E-C-dots suspended in 10 mM potassium phosphate buffer (pH 7.4). Relative to quinine sulfate, quantum yields of B-C-dots and E-C-dots were found to be 4.0% and 5.0%, respectively (Figure S2). These quantum yields are relatively low compared with quantum yields from other bacterially-derived C-dots carbonized using the same method [20,21,22]. However, quantum yields in the literature are obtained in water, while we preferred to measure quantum yields under more physiological conditions, i.e., in a 10 mM phosphate buffer (pH 7.4). C-dots in buffer generally have lower quantum yields [41].

### 3.2. Antibacterial Activity of Hydrothermally Derived Probiotic and Pathogenic C-Dots

Neither probiotic B-C-dots nor pathogenic E-C-dots possessed any growth inhibitory or bactericidal activity towards different strains of planktonic *E. coli* (Table S1), similar to what was observed for *L. plantarum*-derived C-dots that did not inhibit the growth of planktonic *E. coli* [20]. Similar to most C-dots [42,43], our bacterially-derived C-dots generated ROS (see below). However, the short-lived nature of most ROS generally impedes observation of antibacterial activity against planktonic bacteria in suspension [44] due to the large distances that have to be bridged before the ROS generated reaches a target bacterium.

**Figure 4:**
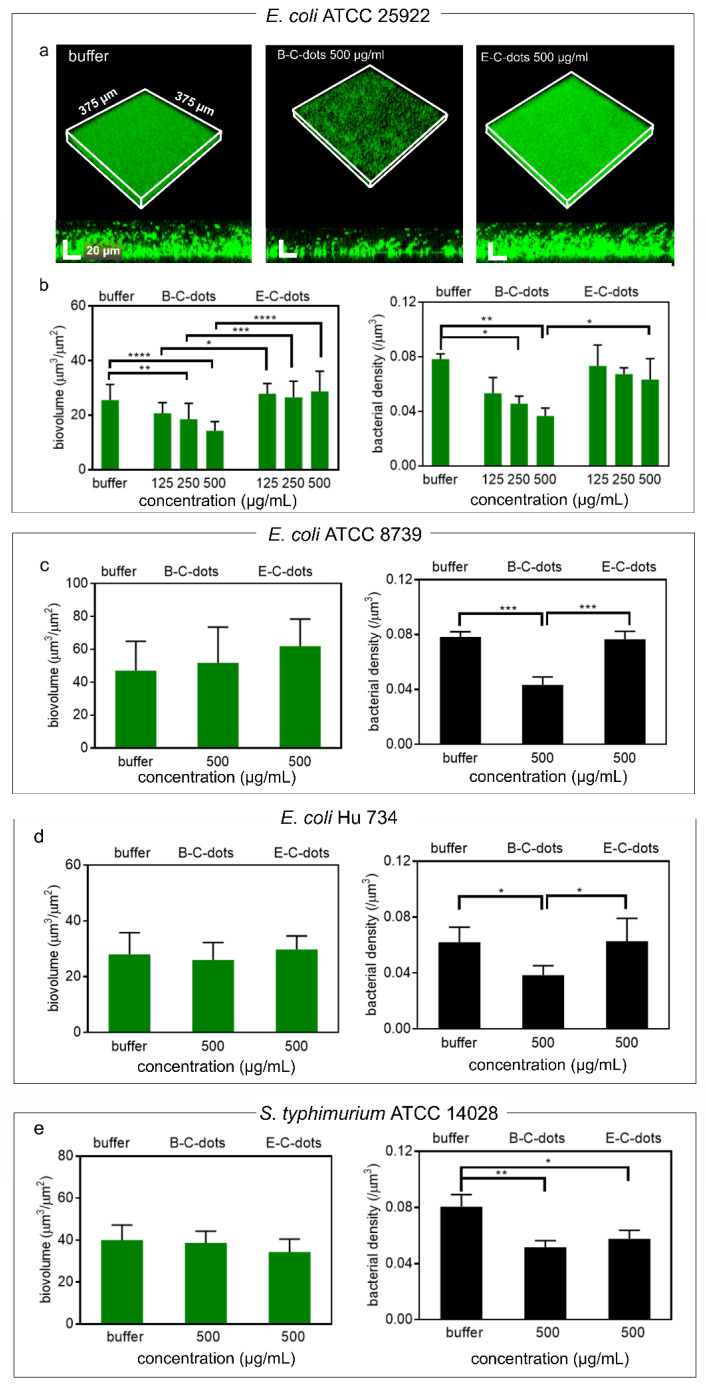
Effects of C-dots hydrothermally derived from *B. breve* ATCC 15700 (B-C-dots) and *E. coli* ATCC 25922 (E-C-dots) on 24 h grown infectious biofilms. (**a**) CLSM images of *E. coli* ATCC 25922 biofilms stained with green-fluorescent SYTO9 and red-fluorescent propidium iodide after 4 h exposure to 10 mM phosphate buffer (pH 7.4), and suspensions of B-C-dots or E-C-dots (500 µg/mL) in phosphate buffer. (**b**) Biovolumes and volumetric bacterial densities of *E. coli* ATCC 25922 biofilms after 4 h exposure to 10 mM phosphate buffer (pH 7.4), and suspensions of B-C- or E-C- dots with different concentrations in phosphate buffer. (**c**) Same as (**b**), but now for *E. coli* ATCC 8739. (**d**) Same as (**b**), but now for *E. coli* Hu 734. (**e**) Same as (**b**), but now for *S. typhimurium* ATCC 14028. All data are expressed as means ± standard deviations over triplicate experiments with separately prepared nanoparticles and different bacterial cultures. * *p* < 0.05, ** *p* < 0.01, *** *p* < 0.001 and **** *p* < 0.0001 indicate significant differences with respect to exposure to buffer or between different C-dot suspensions (one way ANOVA).

Next, the antibacterial activities of the B-C- and E-C-dots were evaluated against biofilms composed of different strains of pathogenic *E. coli* and *S. typhimurium.* Exposure of 24 h old biofilms to 500 µg/mL of B-C-dots or E-C-dots for 4 h yielded no red-fluorescent, membrane damaged *E. coli* upon biofilm staining with SYTO9/propidium iodide (see Figure 4a for example images), in line with the absence of growth inhibitory or bactericidal activity in planktonic bacteria (Table S1). Such as in an aqueous suspension, the distances between bacteria in a biofilm are relatively large for bridging by short-lived ROS, composed of roughly 90% water and extracellular polymeric substances (EPS) [45]. Calculation of the biovolumes of the biofilms after exposure to probiotic B-C-dots from confocal laser scanning microscopical (CLSM) images using ImageJ and COMSTAT [30] demonstrated a small but significant (*p* < 0.05, one way ANOVA) decrease in the biovolumes of biofilms composed of *E. coli* ATCC 25922 from 26 to 14 μm^3^/μm^2^ for buffer and 500 μg/mL B-C-dots, respectively (Figure 4b), but not of biofilms composed of *E. coli* ATCC 8739 (Figure 4c), *E. coli* Hu 734 (Figure 4d) or *S. typhimurium* ATCC 14028 (Figure 4e). However, volumetric bacterial densities (i.e., the number of bacteria per volume unit of biofilm) in biofilms composed of either of the three pathogenic *E. coli* strains or the *S. typhimurium* strain were significantly (*p* < 0.05, one way ANOVA) decreased for 35–50% through exposure to 500 μg/mL B-C-dots compared to buffer (see also Figure 4b–e). This decrease in volumetric density yields more open space in a biofilm for transport of, e.g., antibiotics. Pathogenic E-C-dots neither reduced biovolumes of biofilms composed of their source strain used nor of any other pathogenic strain (see also Figure 4b–e). Pathogenic E-C-dots did not have the ability to reduce volumetric bacterial densities in *E. coli* biofilms while reducing the volumetric bacterial density for *S. typhimurium*. This might be due to structural differences between biofilms of both strains that may be caused by a different EPS composition. FTIR, for instance, has demonstrated that the ratio between proteins and carbohydrates in *E. coli* and Salmonella differs [46]. The lack of growth inhibitory (MIC, Table S1) and bactericidal activity (MBC, see Table S1) of B-C- and E-C-dots, combined with the reductions in volumetric bacterial density in the biofilms observed, led to the conclusion that the matrix of the biofilm must have been disrupted, likely by ROS generated by B-C-dots. Biofilms are, for their major part, composed of water and EPS, which makes it likely that ROS is able to alter the biofilm structure by degrading omnipresent EPS. Accordingly, ROS generation by the different C-dots were compared. Probiotic B-C-dots produced roughly two-fold higher (*p* < 0.05, one-way ANOVA) amounts of ROS (Table S2) than pathogenic E-C-dots. Since ROS generation by C-dots can be due to heterocyclic aromatic amines formed during bacterial carbonization [36,47,48], the higher generation of ROS by B-C-dots can be explained by the higher absorption band ratio at 1590 cm^−1^ due to heterocyclic aromatic amines in the FTIR spectra of B-C-dots as compared with E-C-dots (see also Table 1).

Collectively, it can be concluded that hydrothermally derived probiotic B-C-dots act as a disruptant of *E. coli* and *S. typhimurium* biofilms due to the generation of ROS acting on the biofilm matrix, as an antibacterial inheritance from their probiotic source bacteria. Such an antibacterial inheritance is absent in pathogen-derived E-C-dots.

### 3.3. Synergy between Probiotic or Pathogenic C-Dots and Antibiotics in Eradicating E. coli Biofilm

**Figure 5:**
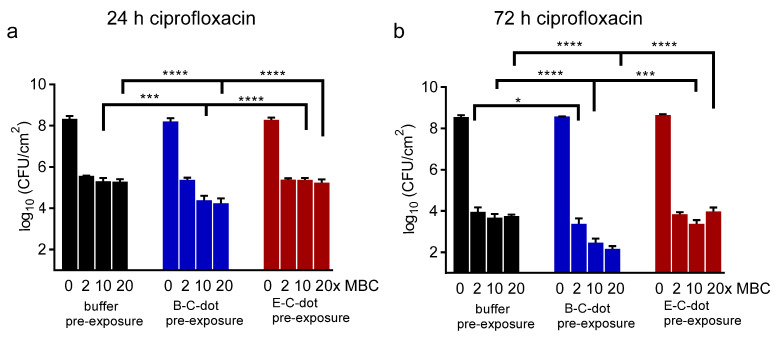
Effects of pre-exposure to C-dots (500 µg/mL) hydrothermally derived from *B. breve* ATCC 15700 (B-C-dots) and *E. coli* ATCC 25922 (E-C-dots) on antibiotic killing in 24 h and 72 h grown *E. coli* biofilm. Exposure to 10 mM phosphate buffer (pH 7.4) was applied as a control. (**a**) Effects of 24 h ciprofloxacin treatment at different concentrations (indicated in multiples of its MBC, see Table S1) on the number of CFUs in *E. coli* ATCC 25922 biofilms. (**b**) Same as panel (**a**), now for 72 h ciprofloxacin treatment. All data are expressed as means ± standard deviations over triplicate experiments with separately prepared C-dots and different *E. coli* cultures. * *p* < 0.05, *** *p* < 0.001 and **** *p* < 0.0001 indicate significant differences with respect to pre-exposure to buffer or different C-dot suspensions (one way ANOVA).

The decrease in the volumetric bacterial density in pathogenic biofilms caused by exposure to probiotic B-C-dots can be expected to yield better penetration of antibiotics into a biofilm and associated bacterial killing by the antibiotic. Accordingly, the pathogen with the highest minimal bactericidal concentration (MBC) against ciprofloxacin, i.e., *E. coli* ATCC 25922 (Table S1), was employed to grow a biofilm that was pre-exposed to C-dots prior to exposure to ciprofloxacin (a common antibiotic used for clinical intestinal infection treatment). Synergistic eradication of *E. coli* ATCC 25922 biofilms pre-exposed to probiotic B-C-dots prior to ciprofloxacin exposure increased when biofilms were pre-exposed to higher concentrations of B-C-dots and after longer antibiotic exposure time (Figure 5a,b). The reduction was 1.2-log when treated with ciprofloxacin at 20 times MBC for 24 h and amounted to 1.6-log when treated at 20 times MBC for 72 h to ciprofloxacin after pre-exposure to B-C-dots. The synergistic effects were only visible for the combination of probiotic B-C-dots with ciprofloxacin but not for its combination with pathogenic E-C-dots, probably due to larger disruption of the biofilm matrix due to two-fold higher ROS generation by probiotic B-C-dots (see Table S2). Therefore, the disruptant properties of probiotic B-C-dots facilitating the better eradication of *E. coli* biofilms by ciprofloxacin is part of the inheritance of antibacterial activities from their probiotic source strain [49].

### 3.4. Biosafety of Hydrothermally Derived Probiotic and Pathogenic C-Dots in Mice

**Figure 6:**
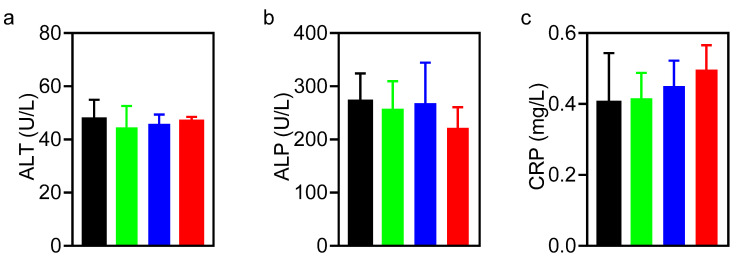

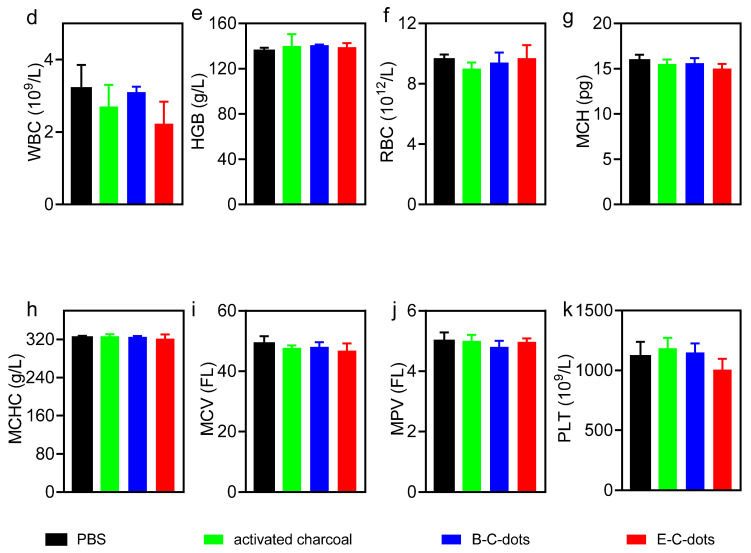
Blood analyses at sacrifice (day 14) of mice after administration of PBS, a commercial activated charcoal product (controls) or hydrothermally derived probiotic B-C-dots or pathogenic E-C-dots (daily dose 0.3 mg/mouse) for 14 days. (**a**) Alanine aminotransferase (ALT). (**b**) Alkaline phosphatase (ALP). (**c**) C-reactive protein (CRP). (**d**) White blood cells (WBC). (**e**) hemoglobin (HGB). (**f**) Red blood cells (RBC). (**g**) Mean corpuscular hemoglobin (MCH). (**h**) Mean corpuscular hemoglobin concentration (MCHC). (**i**) Mean corpuscular volume (MCV). (**j**) Mean platelet volume (MPV). (**k**) Platelets (PLT). Data represent averages over each group of three mice with error bars indicating standard deviations.

Before studying biosafety of probiotic and pathogenic C-dots in mice, it was first established that the exposure of an L929 fibroblast cell line to C-dots had no negative effects on the metabolic activity of the cells in vitro (Figure S3). Next, the biosafety of probiotic and pathogenic C-dots were compared with a commercial charcoal powder in mice over a time period of 14 days. Dosing of the charcoal powder and C-dots were based on the prescribed dose of the commercial product for human consumption after correcting for weight differences between mice and humans. Accordingly, mice were gavaged with a C-dot dose of 0.3 mg/mouse once a day over a period of 14 days. The groups exposed to phosphate-buffered saline (PBS) or activated charcoal were taken as controls. Effective administration and fecal excretion of charcoal powder and C-dots were evidenced by a more blackish or yellowish color of their stool, respectively, as compared with the PBS control group (Figure S4). Body temperature did not change over the course of the experiment (Figure S5a), while the weight of the mice increased at a similar rate in all groups (Figure S5b). In line, after sacrifice at day 14, histological analyses of the heart, liver, spleen, lung and kidneys demonstrated no abnormalities pointing to the presence of C-dots (Figure S6). Blood analyses at sacrifice demonstrated near identical results for all groups with respect to alanine aminotransferase, alkaline phosphatase, C-reactive protein, hemoglobin, red blood cells, mean corpuscular hemoglobin, mean corpuscular hemoglobin concentration, mean corpuscular volume and mean platelet volume (Figure 6). White blood cell counts became 30% lower (Figure 6d), and platelet counts became 10% lower (Figure 6k) in the group of mice that were gavaged with pathogenic E-C-dots, as compared with other groups. C-reactive protein increased from 0.4 to 0.5 mg/mL in the group gavaged with E-C-dots. However, differences were not statistically significant and all within the healthy range. Accordingly, both probiotic and pathogenic C-dots can be considered biosafe in mice.

## 4. Conclusions

Probiotic B-C-dots hydrothermally derived from *B. breve* had inherited more proteins and polysaccharides than pathogenic E-C-dots derived from *E. coli* and contained a new heterocyclic aromatic amine functionality. Unlike pathogenic E-C-dots, probiotic B-C-dots disrupted the infectious biofilm matrix of *E. coli* and *S. typhimurium* strains. Pre-exposure of an *E. coli* biofilm to probiotic B-C-dots synergistically enhanced the killing efficacy of ciprofloxacin better than pre-exposure to pathogenic E-C-dots, due to higher ROS generation causing more extensive disruption of the biofilm matrix. Both types of C-dots can be regarded as biosafe in mice. These conclusions confirm our hypothesis that C-dots hydrothermally derived from probiotic bacteria as a carbon source possess probiotic properties. Currently, orally administered probiotic bacteria need tedious encapsulation in order to provide protection against gastric acids and antibiotics for clinical use in intestinal infection treatment [49,50]. Carbonization of probiotic bacteria is cheap and easy, and probiotic C-dots can be applied as an alternative for probiotic bacteria without additional surface modification, as required for otherwise derived C-dots to act as a biofilm disruptant.

## Figures and Tables

**Table 1:** FTIR absorption band ratios with respect to the C-H absorption band between 2950 and 2850 cm^−1^ and elemental surface compositions derived from XPS spectra for *B. breve* ATCC 15700 and B-C-dots, *E. coli* ATCC 25922 and E-C-dots. FTIR absorption band ratios are averages ± standard deviations over triplicate experiments with separately prepared nanoparticles and different bacterial cultures, while XPS was done in single-fold.

Strain/ C-Dot	FTIR Absorption Band Ratios *				
	**1660 cm^−1^/CH**	**1540 cm^−1^/CH**	**1590 cm^−1^/CH**	**(1260–1245 cm^−1^)/CH**	**(1200–950 cm^−1^)/CH**
*B. breve*	3.4 ± 0.3	0.8 ± 0.1	-	0.7 ± 0.1	3.7 ± 0.2
B-C-dots	1.1 ± 0.5	-	3.2 ± 0.1	0.2 ± 0.1	5.3 ± 1.3
*E. coli*	4.9 ± 0.1	1.7 ± 0.0	-	0.8 ± 0.1	2.0 ± 0.1
E-C-dots	0.8 ± 0.1	-	1.4 ± 0.5	0.1 ± 0.0	2.2 ± 0.4
	XPS Elemental Surface Composition (at%) **				
	C_1s_	N_1s_		O_1s_	P_2p_
*B. breve*	57.6	5.5		32.8	1.1
B-C-dots	57.7	4.9		25.7	2.0
*E. coli*	59.7	7.8		31.1	1.2
E-C-dots	66.6	2.8		22.1	0.3
	Chemical Functionalities Involving C (at%) ***				
	C-C/C=C	C-N/C-O		O=C	-COOH
*B. breve*	13	32		12	1
B-C-dots	37	12		8	1
*E. coli*	20	27		11	1
E-C-dots	47	11		8	0

* Absorption band ratios have been determined after baseline adjustment using Spectragryph software. ** Na, S, Cl, Si were not included in Table 1. *** The percentages of carbon involved in C–C/C=C, C–N/C–O, O=C and –COOH were derived from the decomposition of the C1s peak into four components at 284.8, 286.1, 288.0 and 289.3 eV, respectively. See Figure 2 for corresponding electron binding energy spectra over a narrow binding energy range (276–296 eV).

## Data Availability

Data are available on request.

## Supplementary Materials

The following are available online at https://www.mdpi.com/article/10.3390/pharmaceutics13111809/s1, Figure S1: UV-vis absorption spectra of hydrothermally derived probiotic B-C- and pathogenic E-C-dots in 10 mM phosphate buffer at pH 7.4, Figure S2: Integrated fluorescence intensity as a function of absorbance of hydrothermally derived B-C- and E-C-dots at pH 7.4 in 10 mM phosphate buffer. The ratio of absorption to emission was derived by linear interpolation (drawn lines), while calculating the quantum yield from the slopes relative to the slope for quinine sulfate with a known quantum yield (54%), Figure S3: Viability of L929 mouse fibroblasts after 24 h exposure to different concentrations (µg/mL) of hydrothermally derived, probiotic B-C- or pathogenic E-C-dots. Viability is expressed as a percentage of the XTT conversion of cells exposed to growth medium. All data represent means ± SD over triplicate experiments with separately prepared nanoparticles, Figure S4: Stool color of mice during administration of phosphate buffered saline (PBS), a commercial activated charcoal product or hydrothermally derived probiotic B-C- or pathogenic E-C-dots (daily dose 0.3 mg/mouse) during 14 days. Scale bar denotes 1 cm, Figure S5: (a) Body temperature of mice during administration of PBS, a commercial activated charcoal product (controls) or hydrothermally derived probiotic B-C-dots or pathogenic E-C-dots (daily dose 0.3 mg/mouse) during 14 days. (b) Same as panel a, now for body weight. Data represent averages over each group of three mice with error bars indicating standard deviations, Figure S6: Histology of major organs after H&E staining of mice at sacrifice (day 14) after administration of PBS, a commercial, activated charcoal product (controls) or hydrothermally derived probiotic B-C-dots or pathogenic E-C-dots (daily dose 0.3 mg/mouse) during 14 days, including the heart, liver, spleen, lungs, and kidneys. Scale bar equals 100 µm, Table S1: Minimum inhibitory (MIC) and Minimal bactericidal (MBC) concentrations of the *E. coli* and *S. typhimurium* strains employed for probiotic B-C- and pathogenic E-C-dots and ciprofloxacin, as measured in 100% BHI for *E. coli* strains and NB for *S. typhimurium*, Table S2: Generation of reactive oxygen species (ROS) by hydrothermally derived, probiotic B-C- or pathogenic E-C-dots in 10 mM potassium phosphate buffer at pH 7.4. ROS generation is expressed in arbitrary units, obtained from a DCFH-based assay.
