# Supplementary material for: Synergy between “Probiotic” Carbon Quantum Dots and Ciprofloxacin in Eradicating Infectious Biofilms and Their Biosafety in Mice
Source: Pharmaceutics. 2021 Oct 29;13(11):1809. doi: 10.3390/pharmaceutics13111809 (PMC8620463; doi:10.3390/pharmaceutics13111809)
Supplement: Supplementary file 1 [file pharmaceutics-13-01809-s001.zip › pharmaceutics-1409202-supplementary.pdf]

pharmaceutics

Supplementary Materials: Synergy between "Probiotic" Carbon Quantum Dots and Ciprofloxacin in Eradicating Infectious Biofilms and Their Biosafety in Mice

Yanyan Wu, Guang Yang, Henny C. van der Mei, Linqi Shi, Henk J. Busscher and Yijin Ren

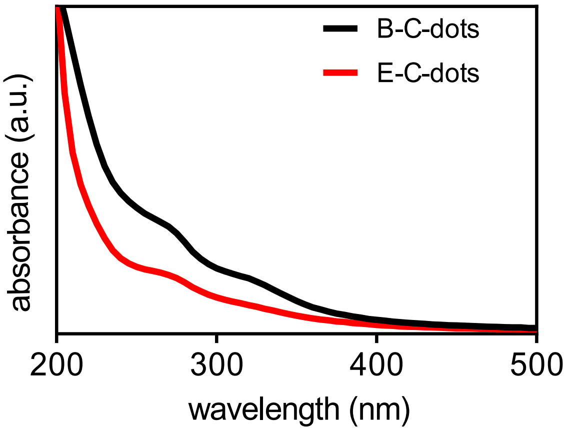

Figure S1. UV-vis absorption spectra of hydrothermally derived probiotic B-C- and pathogenic E-C-dots in 10 mM phosphate buffer at pH 7.4.

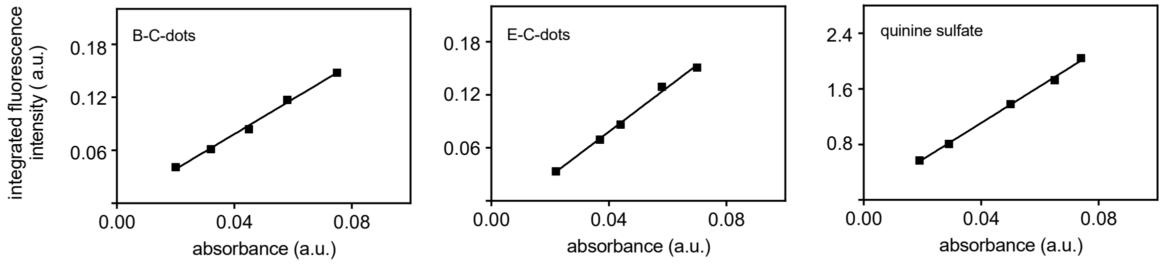

Figure S2. Integrated fluorescence intensity as a function of absorbance of hydrothermally derived B-C- and E-C-dots at pH 7.4 in 10 mM phosphate buffer. The ratio of absorption to emission was derived by linear interpolation (drawn lines), while calculating the quantum yield from the slopes relative to the slope for quinine sulfate with a known quantum yield (54%).

Table S1. Minimum inhibitory (MIC) and Minimal bactericidal (MBC) concentrations of the *E. coli* and *S. typhimurium* strains employed for probiotic B-C- and pathogenic E-C-dots and ciprofloxacin, as measured in 100% BHI for *E. coli* strains and NB for *S. typhimurium*.

**Material**	***E. coli* ATCC 25922**		***E. coli* ATCC 8739**		***E. coli* Hu 734**		***S. typhimurium* ATCC 14028**	
	MIC (µg/mL)	MBC (µg/mL)	MIC (µg/mL)	MBC (µg/mL)	MIC (µg/mL)	MBC (µg/mL)	MIC (µg/mL)	MBC (µg/mL)
B-C-dots	>1000	>1000	>1000	>1000	>1000	>1000	>1000	>1000
E-C-dots	>1000	>1000	>1000	>1000	>1000	>1000	>1000	>1000
Ciprofloxacin	0.16	1.25	0.04	0.08	0.04	0.62	0.16	0.31

Table S2. Generation of reactive oxygen species (ROS) by hydrothermally derived, probiotic B-C- or pathogenic E-C-dots in 10 mM potassium phosphate buffer at pH 7.4. ROS generation is expressed in arbitrary units, obtained from a DCFH-based assay.

Carbon dots	B-C-dots	B-C-dots with DCFH	E-C-dots	E-C-dots with DCFH
Fluorescence intensity (a.u.)	3.8 ± 0.6	71.4 ± 7.4	2.5 ± 0.3	43.6 ± 5.8

*All data are expressed in arbitrary units as means ± standard deviations over triplicate experiments with separately prepared nanoparticles.

Method: ROS generation by the C-dots was determined by oxidation of non-fluorescent 2′,7′-dichlorofluorescein (DCFH) to highly fluorescent 2′,7′-dichlorofluorescein (DCF). First, bacteria-free DCFH was prepared [1,2] by mixing 10 mg/mL DCFDA dissolved in dimethylsulfoxide for 30 min in the presence of 0.01 M NaOH in the dark at room temperature. Next, the mixture was neutralized with potassium phosphate buffer pH 7.4 to obtain a final concentration of 10 µg/mL DCFH. Freshly prepared C-dot suspensions (200 µL, 500 µg/mL) were put in 96-wells plates, 10 µL DCFH was added and the mixture was kept in the dark for 4 h at 37°C and fluorescence intensity was measured using a spectrophotometer (Synergy H1, BioTek, USA) at excitation and emission wavelengths of 500 and 535 nm, respectively. Fluorescence intensity of DCFH dissolved in potassium phosphate buffer without C-dots amounted 4.9±0.7.

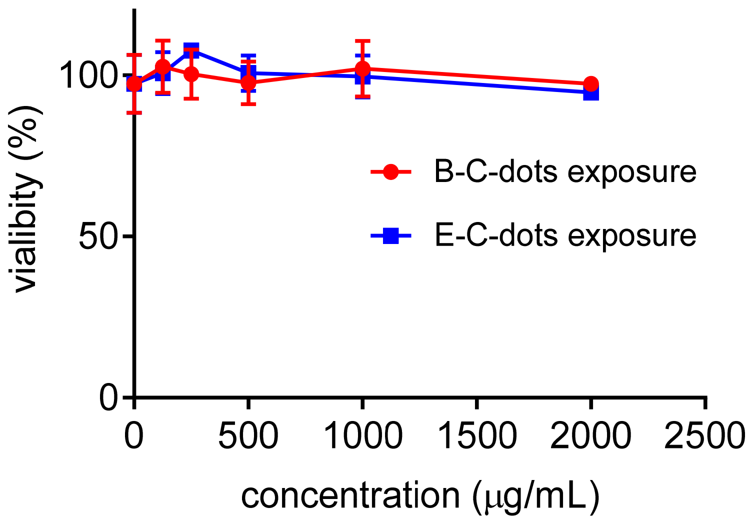

Figure S3. Viability of L929 mouse fibroblasts after 24 h exposure to different concentrations (µg/mL) of hydrothermally derived, probiotic B-C- or pathogenic E-C-dots. Viability is expressed as a percentage of the XTT conversion of cells exposed to growth medium. All data represent means ± SD over triplicate experiments with separately prepared nanoparticles.

Method: Viability of mouse fibroblasts (L929) was measured using an XTT (2,3-bis[2-methoxy-4-nitro-5-sulfophenyl]-2H-tetrazolium-5-carboxyanilide inner salt) assay. Fibroblasts were cultured in 96-wells plates (2 × 10^5 cells/well) in Eagle minimum essential medium supplemented with 10% fetal bovine calf serum and 1% penicillin/streptomycin. Hydrothermally derived B-C-dots or E-C-dots suspended in growth medium were added to the wells at different concentrations (125, 250, 500, 1000, 2000 µg/mL). After 24 h incubation, at 37°C in a 5% CO_2 atmosphere, 20 µL of XTT containing phenazine methosulfate was added to each well. After 3 h incubation, absorbances at a wavelength of 480 nm were

measured using a spectrophotometer (Synergy H1, BioTek, USA). Absorbances were expressed as a percentage conversion relative to fibroblasts exposed to growth medium (0 µg/mL C-dots), set at 100%.

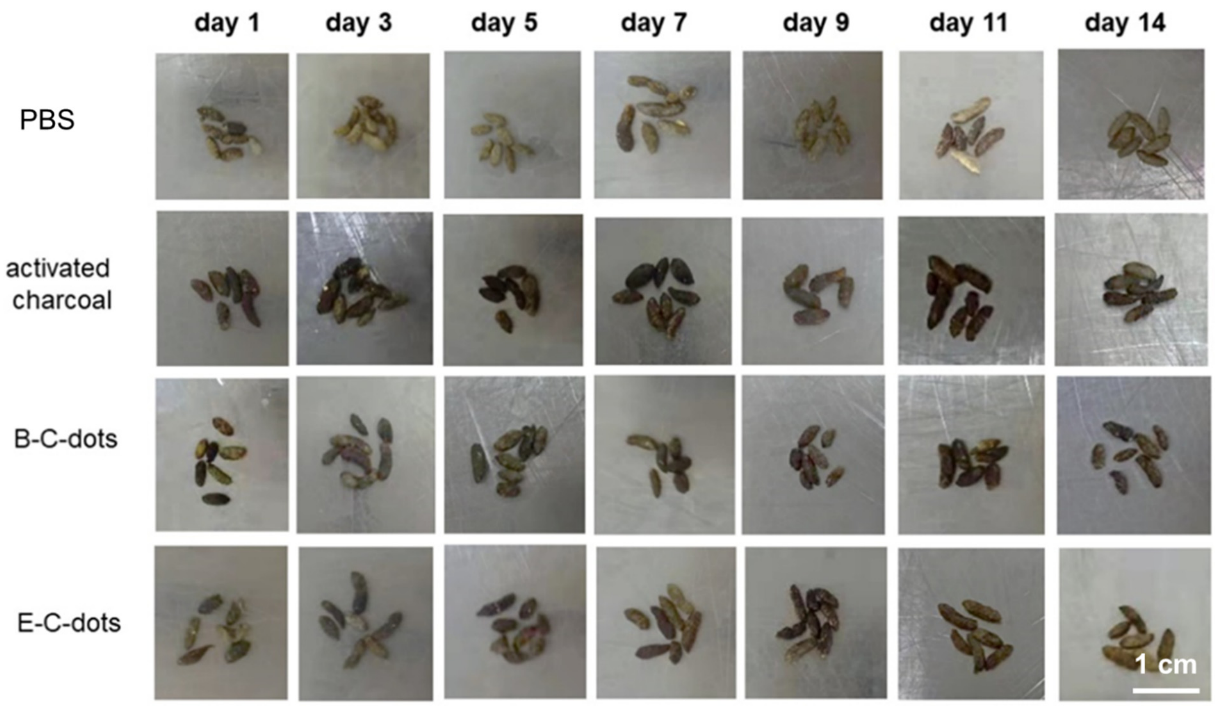

Figure S4. Stool color of mice during administration of phosphate buffered saline (PBS), a commercial activated charcoal product or hydrothermally derived probiotic B-C- or pathogenic E-C-dots (daily dose 0.3 mg/mouse) during 14 days. Scale bar denotes 1 cm.

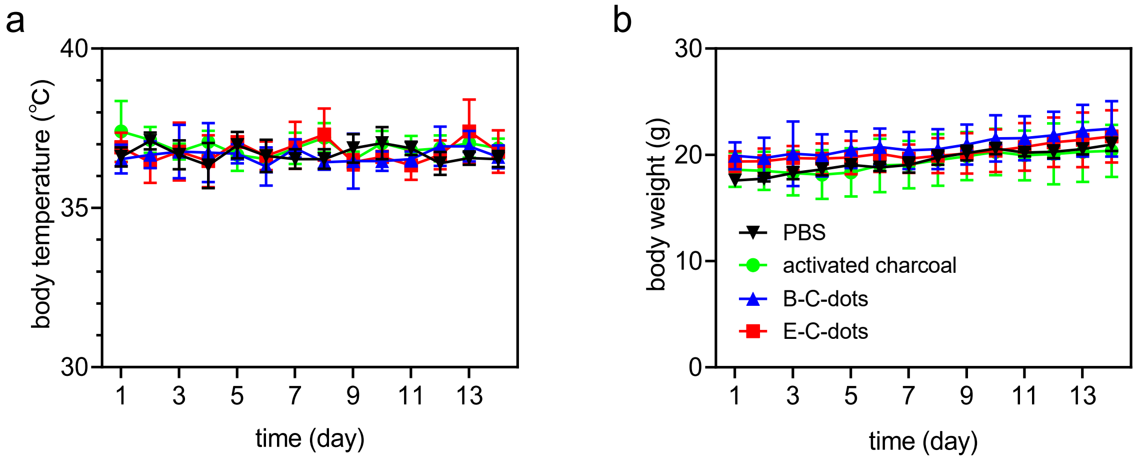

Figure S5. (a) Body temperature of mice during administration of PBS, a commercial activated charcoal product (controls) or hydrothermally derived probiotic B-C-dots or pathogenic E-C-dots (daily dose 0.3 mg/mouse) during 14 days. **(b)** Same as panel a, now for body weight. Data represent averages over each group of three mice with error bars indicating standard deviations.

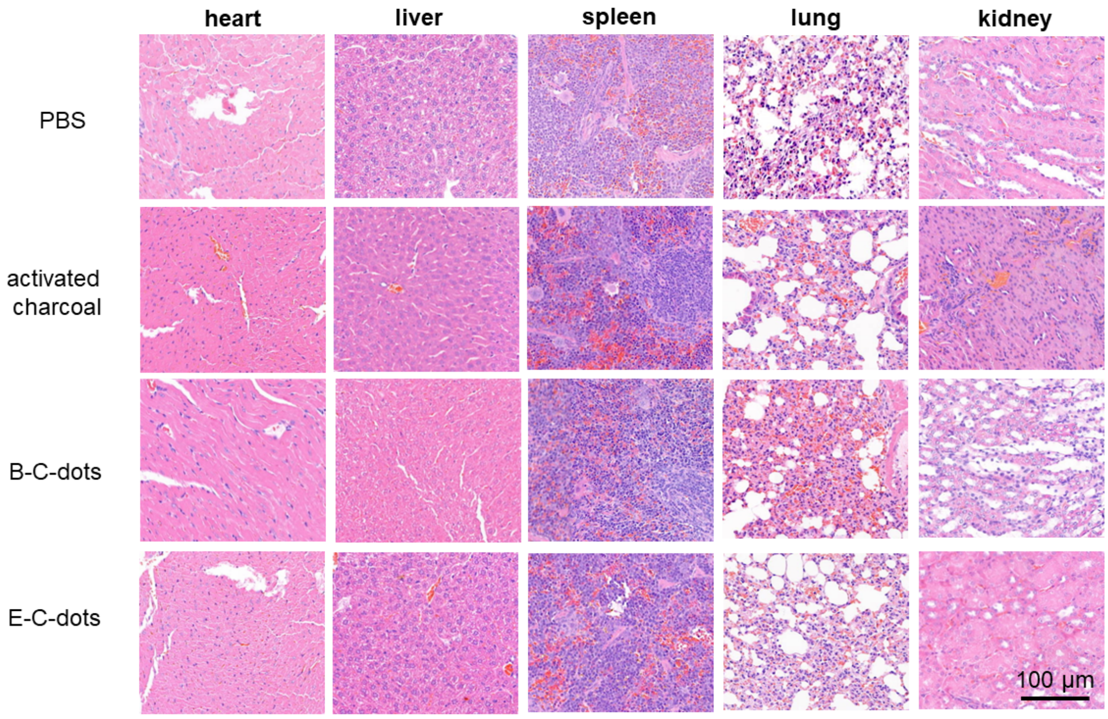

Figure S6. Histology of major organs after H&E staining of mice at sacrifice (day 14) after administration of PBS, a commercial, activated charcoal product (controls) or hydrothermally derived probiotic B-C-dots or pathogenic E-C-dots (daily dose 0.3 mg/mouse) during 14 days, including the heart, liver, spleen, lungs, and kidneys. Scale bar equals 100 μm.

Method: Organs were surgically collected and cut in 3–5 mm pieces that were fixed in 10% formaldehyde for 24 h at 4°C and embedded into paraffin. 5 μm thick sections were obtained after dehydration and H (hematoxylin) & E (eosin) stained. Stained sections were examined using an optical microscope (Leica DM4000M, Wetzlar, Germany).

References

1. Alekseenko, A. V.; Waseem, T. V.; Fedorovich, S. V. Ferritin, a protein containing iron nanoparticles, induces reactive oxygen species formation and inhibits glutamate uptake in rat brain synaptosomes. *Brain Res.* **2008**, *1241*, 193–200.
2. LeBel, C.P.; Bondy, S.C. Sensitive and rapid quantitation of oxygen reactive species formation in rat synaptosomes. *Neurochem. Int.* **1990**, *17*, 435–440.
